# The Brief Memory and Executive Test (BMET) for detecting vascular cognitive impairment in small vessel disease: a validation study

**DOI:** 10.1186/s12916-015-0290-y

**Published:** 2015-03-11

**Authors:** Rebecca L Brookes, Matthew J Hollocks, Usman Khan, Robin G Morris, Hugh S Markus

**Affiliations:** Stroke and Dementia Research Centre, St George’s, University of London, Cranmer Terrace, London, SW17 0RE UK; Department of Clinical Neurosciences, University of Cambridge, R3, Box 183, Addenbrookes Biomedical Campus, Cambridge, CB2 0QQ UK; Department of Psychology, King’s College London, Institute of Psychiatry, Psychology and Neuroscience, PO Box 078, De Crespigny Park, London, SE5 8AF UK

**Keywords:** Cognitive disorders, Executive function, Memory, Vascular dementia

## Abstract

**Background:**

Cognitive impairment is common in patients with cerebral small vessel disease, but is not well detected using common cognitive screening tests which have been primarily devised for cortical dementias. We developed the Brief Memory and Executive Test (BMET); a rapid screening measure sensitive to the impaired executive function and processing speed characteristic of small vessel disease (SVD). To assess the BMET’s validity for general use, we evaluated it when administered by non-psychologists in a multicentre study and collected control data to derive normative scores.

**Methods:**

Two-hundred participants with SVD, defined as a clinical lacunar stroke and a corresponding lacunar infarct on MRI, and 303 healthy controls aged between 40–90 years old were recruited. The BMET, as well as the Montreal Cognitive Assessment (MoCA) and Mini Mental State Examination (MMSE), were performed. Overall, 55 SVD participants underwent repeat testing at 3 months to assess the BMET test-retest reliability.

**Results:**

Administering the BMET took a mean (SD) of 12.9 (4.7) in cases and 9.5 (2.6) minutes in controls. Receiver Operator Curve analysis showed the BMET was a good predictor of cognitive impairment in SVD (AUC = 0.94) and performed significantly better than both the MoCA (AUC = 0.77) and the MMSE (AUC = 0.70). Using a cut-off score of 13, the BMET had a sensitivity of 93% and specificity of 76% for detecting cognitive impairment in SVD.

**Conclusions:**

The BMET is a brief and sensitive tool for the detection of cognitive impairment in patients with SVD.

**Electronic supplementary material:**

The online version of this article (doi:10.1186/s12916-015-0290-y) contains supplementary material, which is available to authorized users.

## Background

Cerebral small vessel disease (SVD), which causes lacunar stroke accounting for 10 to 30% of all ischemic strokes, is the major cause of vascular cognitive impairment (VCI) and vascular dementia [1]. VCI due to SVD is associated with a characteristic profile of impairment in cognitive flexibility, attention, and processing speed with a relatively spared performance on memory tasks [2]. Cognitive impairment (CI) in patients presenting with lacunar stroke or other manifestations of SVD is common [3], with up to 50% of patients with lacunar stroke having some degree of cognitive impairment, which predicts subsequent progression to vascular dementia [4,5], and has been shown to have a major impact on quality of life [6]. However, despite this, VCI associated with SVD is often missed in a clinical setting.

A key contributing factor to under diagnosis is the current use of cognitive screening measures, such as the Mini Mental State Examination (MMSE) [7], that have been developed primarily for the assessment of cortical dementias such as Alzheimer’s Disease and have been shown to be insensitive to the cognitive deficits found in patients with VCI due to SVD [8]. The lack of a widely available validated screening tests for this group has been recognised in the National Institute of Neurological Disorders and Stroke-Canadian Stroke Network Vascular Cognitive Impairment Harmonization Standards [9], which proposed specific cognitive protocols, but these take a minimum of 20 to 60 minutes to complete. The Brief Memory and Executive Test (BMET) [10] was designed to provide a cognitive screen taking approximately 10 minutes, which would be suitable for use by health professionals in in- and out-patient settings. It is primarily for screening VCI in patients with SVD, helping differentiate such deficits from those found in patients with cortical dementias; it incorporates specifically adapted measures of executive functioning and processing speed, designed to be brief but sensitive, combined with measures of memory functioning. In a preliminary study, the BMET accurately differentiated VCI due to SVD from CI due to Alzheimer’s disease, with a sensitivity of 91% and specificity of 85% [10].

We performed a comprehensive validation of the previously developed BMET [10], evaluating it in 19 stroke centres throughout England, with administration of the BMET by doctors and research nurses rather than neuropsychologists. We also recruited a large control sample to standardise the test and compared the performance of the BMET against the Montreal Cognitive Assessment (MoCA [11]) and the Mini Mental State Examination (MMSE [7]), two measures frequently used as brief cognitive screening tools in stroke patients, including patients with SVD.

## Methods

This study was approved by the London Bridge Research Ethics Committee (11/LO/0636) and all participants gave informed consent before taking part in this study.

### Participants

Two-hundred participants with lacunar stroke were recruited from 19 sites across the English Stroke Research Network (see below for a list of sites). In order to match SVD patients with controls, we included all participants aged between 40 and 90 years of age (n = 196), who were fluent in English. All participants were tested >3 months post-stroke to exclude any acute effects of stroke on cognitive performance. The sample size was calculated based on a meta-analysis examining studies looking at the relationship between leukoaraiosis and cognitive performance [12], which found modest correlations of r = 0.2 to 0.3. Taking the mid-point (r = 0.25), we calculated that a sample of 200 would produce acceptable significance levels for a range of analyses, including those correcting for multiple comparisons using conservative measures such as a Bonferroni correction. All patients included in the study presented with a clinical lacunar syndrome (e.g., hemiparesis, hemisensory deficit, sensorimotor deficit, ataxic hemiparesis, or clumsy hand dysarthria) or partial lacunar syndrome (e.g., pure motor stroke affecting face and arm or arm and leg) with an MRI confirmed lacunar infarct in an anatomically corresponding location. Lacunar infarction was defined as a subcortical infarct ≤1.5 cm in diameter, on MRI.

For all cases, MRI scans were centrally reviewed to confirm eligibility and to grade the degree of leukoaraiosis using the Fazekas’ scale [13]. Exclusion criteria were as follows: i) stenosis >50% in the extracranial or intracranial cerebral vessels, or previous carotid endarterectomy, ii) cardioembolic source of stroke, defined according to the TOAST criteria as high or moderate probability, and/or iii) the presence of a cortical infarct >1 cm diameter on MRI. SVD patients were included if they had either isolated lacunar infarcts (n = 122) or lacunar infarcts with leukoaraiosis (Fazekas grade ≥2, n = 74), the lacunar infarcts occurring for the first time or recurrently.

Overall, 303 healthy controls were recruited from local family doctors practices or other volunteer groups. Individuals with cardiovascular risk factors and other comorbidities were included, but individuals with a past history of stroke, transient ischemic attack, and major central neurological or major psychiatric disease were excluded. Controls were recruited in stratified age bands to provide a representative sample. In order to establish the test-retest reliability of the BMET, 55 of the SVD patients and 105 controls were retested after 3 months.

### Measures

The Brief Memory and Executive Test (BMET) incorporates tests divided into two main categories: i) executive functioning and processing speed which includes letter-number matching, motor-sequencing, letter-sequencing, and number-letter sequencing; and ii) orientation and memory which includes orientation, five-item repetition, five-item recall, and five-item recognition memory. Full descriptions of each task are presented in the supplementary materials (see Additional file 1). Two versions of the BMET were created, one for right-handed and another for left-handed patients. The difference here is that that the sequencing measures were mirrored to reduce covering of the letters/numbers during test completion. The BMET is available to download from the Cambridge University Stroke Research Group [14].

In addition, we collected data for a range of descriptive variables and background or comparative standardized measures, including the National Adult Reading Test (NART) [15], which is a commonly used and validated measure of pre-morbid intellectual ability, MMSE [7] and MoCA [11] (see Table 1 for descriptive statistics).

**Table 1:** **Descriptive statistics of demographic and clinical variables for participants with small vessel disease and controls**

**Measure**	**Control (n = 303)**	**SVD (n = 196)**	***t*** **-test**
**Demographic variables, Mean (SD)**			
Age	62.5 (13.8)	63.5 (9.9)	*t* = 0.86 (*P* = 0.38)
Years education	14.0 (2.8)	13.7 (3.8)	*t* = 1.2 (*P* = 0.22)
Gender (% male)	54%	68%	
**Socioeconomic status**			
Professional	13%	11%	χ^2^ = 0.50 (*P* = 0.47)
Managerial	42%	34%	χ^2^ = 3.9 (*P* = 0.05)
Skilled	35%	40%	χ^2^ = 1.08 (*P* = 0.29)
Partly skilled	7%	10%	χ^2^ = 1.25 (*P* = 0.26)
Unskilled	1%	2%	χ^2^ = 0.95 (*P* = 0.32)
Other	2%	3%	χ^2^ = 1.2 (*P* = 0.27)
**Risk factors, Mean (SD)**			
Weight (kg)	74.8 (18.3)	81.6 (15.9)	*t* = 4.3 (*P* ≤0.001)
Systolic BP (mmHg)	132 (17.7)	139.7 (17.9)	*t* = 4.4 (*P* ≤0.001)
Diastolic BP (mmHg)	78.9 (10.7)	80.6 (10.5)	*t* = 1.6 (*P* = 0.102)
Units of alcohol per week	11.6 (13.1)	8.54 (14.6)	*t* = 1.2 (*P* = 0.016)
Time since last stroke (months)	–	20.5 (32.3)	–
Treated hypertension	28%	75%	χ^2^ = 103 (*P* ≤0.001)
Treated hyperlipidaemia	24%	78%	χ^2^ = 143 (*P* ≤0.001)
Diabetes mellitus (%)	6%	23%	χ^2^ = 30.3 (*P* ≤0.001)
Smoker (% ever a smoker)	58%	44%	χ^2^ = 19.4 (*P* ≤0.001)
**Cognitive scores**			
MMSE	28.2 (1.9)	28.04 (2.2)	*t* = 1.1 (*P* = 0.27)
MoCA	25.6 (3.2)	24.7 (3.3)	*t* = 2.8 (*P* = 0.005)

MoCA, Montreal Cognitive Assessment; MMSE, Mini Mental State Examination.

### Statistical analysis

Differences between the two main groups on descriptive variables, the BMET raw scores, and other cognitive tests were explored using *t*-tests and χ^2^ tests. Normative data from the control sample was then used to establish age-scaled scores for each of the BMET tests. To do this, the control sample was divided into seven age groups: 40–49 (n = 72), 50–59 (n = 71), 60–69 (n = 49), 70–74 (n = 30), 75–79 (n = 37), 80–84 (n = 36), and 85–90 (n = 8). Based on the mean and standard deviation (SD) for each individual task within each age group, cut-off scores were established for the assignment of scores of “1” for those who scored between 1 or 2 SD’s below the normative mean and “0” for those who scored 2 SD’s or more below the normative mean. A score of “2” was assigned to participants who scored within 1 SD of the normative mean. This allowed the generation of an age-scaled total score (0–16), an executive functioning/processing speed sub-scale (0–8), and an orienting/memory subscale (0–8). The internal consistency and test-retest reliability of these index scores were tested using the Cronbach’s alpha (α) test and correlations, respectively.

VCI was then defined within the SVD sample using the pre-defined Petersen Mild Cognitive Impairment threshold [16] of scoring ≤1.5 SD of the control population mean on a given test. We refined this to make it more conservative by making a classification of VCI where patients fell below this threshold on at least four of eight of the BMET tests. This was the same definition used in the preliminary BMET study [10].

This method stratified the SVD group into those who did (SVD-CI, n = 26) or did not (SVD-nCI, n = 170) meet criteria for VCI. Group differences in cognitive performance between these resulting groups (raw scores) were compared using a non-parametric Wilcoxon Signed-rank test due to the unbalanced group sizes (these groups did not significantly differ on descriptive variables apart from a trend for SVD-CI to have higher systolic blood pressure (z = 1.8, *P* = 0.07)).

Finally, relative sensitivity and specificity of the BMET, MMSE, and MoCA to identify CI in SVD was explored by plotting a receiver operating characteristic (ROC) curve for each measure and comparing the area under the curve (AUC) for each. AUC is a standard measure of the strength of a test with a score of 1.0 representing the best discriminant ability and 0.5 being at chance level. The current analysis consisted of a logistic regression where the relative cognitive tests’ total score (i.e., BMET, MoCA, or MMSE) was regressed onto a dichotomous variable of the presence or absence of cognitive impairment to establish whether it was a significant predictor of group status. This was followed by the plotting of a ROC curve and the generation of optimal cut-off points for the BMET using the *roctab* command in STATA. Finally, the AUCs for each measure were compared using the *roccomp* command.

In order to ensure that we did not inadvertently inflate the AUC of the BMET by defining CI using this test alone, we performed a secondary analysis using a more stringent cut-off for VCI diagnosis. This categorised patients as having VCI if they scored ≤1.5 SD of the control population mean on any four of the BMET subtests, and in addition met the MoCA cut-off for CI (<26, n = 20).

All analyses were conducted in STATA 13 [17].

## Results

### Descriptive statistics

The SVD and control groups did not differ significantly in age, years of education, or IQ (NART), and were closely matched on socioeconomic status. Demographics of both groups are shown in Table 1. Review of the MRI scans revealed that 59% of the SVD patients had isolated lacunar infarcts without leukoaraiosis, 37.5% had multiple lacunar infarcts and confluent leukoaraiosis, and 3.5% had multiple lacunar infarcts without leukoaraiosis. On Fazekas scale grading 38% scored ≥2, 15% scored 1, and 47% scored 0. Missing data for key variables were as follows: height/weight, SVD = 1, control = 6; blood pressure, SVD = 11, control = 0.

### Cognition on MOCA and MMSE

The SVD group performed worse than controls on the MoCA (SVD, mean = 24.7, SD = 3.3; control, mean = 25.6, SD = 3.2, independent *t*-test, *P* = 0.005). In contrast, the groups did not differ on the MMSE (SVD, mean = 28.04, SD = 2.2; control, mean = 28.2, SD = 1.9, *P* = 0.27).

### BMET Performance

The mean (SD) time taken to administer the BMET was 12.9 (4.7) minutes in SVD cases and 9.5 (2.6) minutes in controls.

The SVD group performed worse than the controls on all BMET raw scores except orientation (Independent *t*-tests, see Table 2); this subtest was included to help discriminate SVD from early Alzheimer’s disease [10]. Table 3 shows the age-scaled test scores and indices based on control group data, and shows the SVD-CI group scoring significantly lower on total performance, the executive/processing speed index, the orientation/memory index scores, and all of the individual subtests when compared to the SVD-nCI group.

**Table 2:** **Group differences in performance on the Brief Memory and Executive Test sub-scale raw scores (mean, SD)**

	**Control**	**SVD**	***t*** **-test**
Time taken (minutes)	9.5 (2.6)	12.9 (4.7)	*t* = 10.3 (*P* ≤0.001)
**Executive functioning and processing speed**			
Letter-number matching (0–40)	27.7 (7.0)	24.1 (7.7)	*t* = 5.3 (*P* ≤0.001)
Motor sequencing (s)	10.7 (8.0)	21.4 (22.1)	*t* = 7.2 (*P* ≤0.001)
Letter sequencing (s)	36.8 (24.7)	56.1 (39.6)	*t* = 6.7 (*P* ≤0.001)
N-L sequencing (s)	49.2 (43.3)	73.0 (57.3)	*t* = 5.3 (*P* ≤0.001)
**Orientation and memory**			
Orientation (0–10)	9.90 (0.3)	9.87 (0.4)	*t* = 0.73 (*P* = 0.46)
Five item repetition (0–15)	14.5 (1.1)	13.8 (1.9)	*t* = 5.1 (*P* ≤0.001)
Five item recall (max 5)	3.7 (1.4)	3.04 (1.7)	*t* = 5.8 (*P* ≤0.001)
Five item recognition (max 5)	4.2 (1.0)	3.7 (1.6)	*t* = 5.1 (*P* ≤0.001)

**Table 3:** **Brief Memory and Executive Test (BMET) normalised individual, total performance, and index scores for the whole Small Vessel Disease (SVD) group and for those with and without cognitive impairment**

	**SVD (whole sample)**	**SVD-nCI (n = 170)**	**SVD-CI (n = 26)**	**SCD-nCI vs. SVD-CI**
**Executive functioning and processing speed**				
Letter-number matching	1.7 (0.6)	1.8 (0.5)	1.1 (0.7)	z = 5.9 (*P* ≤0.001), r = 0.42
Motor sequencing	1.7 (0.7)	1.8 (0.6)	1.1 (0.9)	z = 4.6 (*P* ≤0.001), r = 0.33
Letter sequencing	1.7 (0.7)	1.9 (0.5)	0.6 (0.8)	z = 8.7 (*P* ≤0.001), r = 0.62
N-L sequencing	1.8 (0.6)	1.9 (0.4)	0.9 (0.8)	z = 7.9 (*P* ≤0.001), r = 0.56
**Orientation and Memory**				
Orientation	1.9 (0.4)	1.9 (0.4)	1.7 (0.6)	z = 2.3 (*P* = 0.02), r = 0.16
Five item repetition	1.5 (0.8)	1.7 (0.7)	0.6 (0.9)	z = 5.8 (*P* ≤0.001), r = 0.41
Five item recall	1.7 (0.7)	1.8 (0.6)	1.2 (0.9)	z = 3.7 (*P* ≤0.001), r = 0.25
Five item recognition	1.7 (0.7)	1.8 (0.6)	1.4 (0.9)	z = 2.4 (*P* = 0.02), r = 0.17
**Composite total score and sub-scales**				
BMET Total	13.6 (2.9)	14.4 (1.9)	8.7 (2.8)	z = 7.4 (*P* ≤0.001), r = 0.53
BMET EF/PS	6.9 (1.8)	7.3 (1.2)	3.8 (2.1)	z = 7.9 (*P* ≤0.001), r = 0.56
BMET Orient/Mem	6.7 (1.6)	7.1 (1.3)	4.9 (1.9)	z = 5.6 (*P* ≤0.001), r = 0.40

SVD-nCI, SVD with no cognitive impairment; SVD-CI, SVD with cognitive impairment.

### Internal consistency and test-retest reliability of the BMET

Internal consistency was found to be adequate for total performance (whole sample, α = 0.82; SVD, α = 0.69; control, α = 0.61) and the executive functioning/processing speed index (whole sample, α = 0.78; SVD, α = 0.72; control, α = 0.60), while the internal consistency for the orientation/memory index was considerably lower (whole sample, α = 0.68; SVD, α = 0.35; control, α = 0.45). Test-retest reliability in the SVD subgroup (n = 54) over a 3 month period was also found to be adequate for total performance (r = 0.69, *P* ≤0.001), executive functioning/processing speed (r = 0.68, *P* ≤0.001), and orientation/memory (r = 0.40, *P* ≤0.001). Similar results were found for the control group for total performance (r = 0.47, *P* ≤0.001), executive functioning/processing speed (r = 0.61, *P* ≤0.001), and orientation/memory (r = 0.40, *P* ≤0.001).

### ROC analysis of the BMET and comparison with the MoCA and MMSE

Using our predefined criteria (see Methods), approximately 13% of participants with SVD had VCI. We calculated the sensitivity and specificity of the BMET, MoCA, and MMSE to VCI, and then plotted ROC curves for each measure.

**Figure 1:**
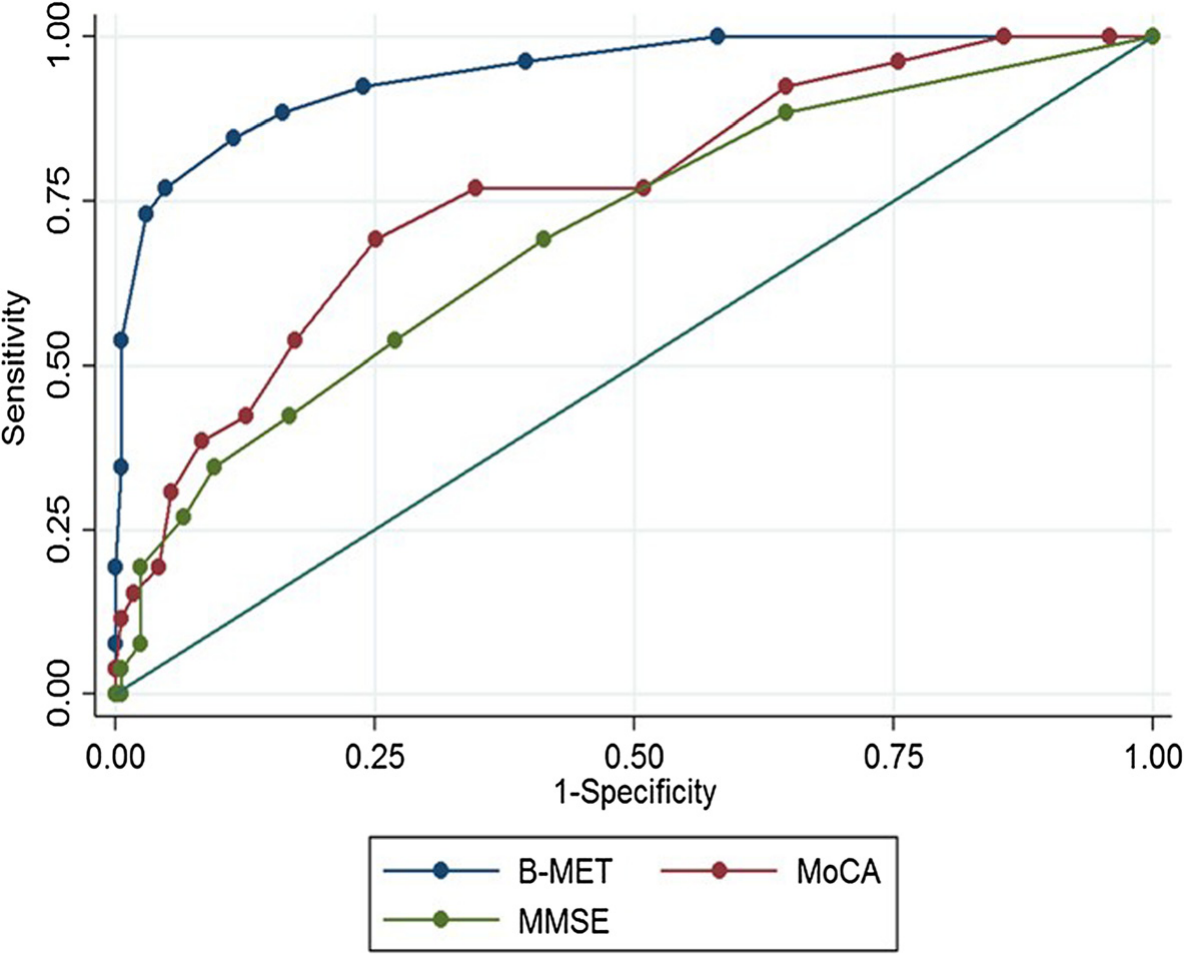
**Comparison of ROC analysis of the Brief Memory and Executive Test, Montreal Cognitive Assessment, and Mini Mental State Examination tests of cognitive impairment.**

The BMET total performance index was a significant predictor of group status (β = 0.79, odds ratio (OR) = 2.22, *P* ≤0.001), with the ROC analysis indicating an AUC of 0.94 (95% confidence interval (CI) = 0.89–0.99). From the ROC curve, an optimal BMET total cut-off score of “13” was identified, which identified VCI in SVD with 93% sensitivity and 76% specificity, correctly classifying 78% of patients. Additional file 2 provides a table containing the sensitivity and specificity for alternative BMET cut-off’s and their comparison with MoCA and MMSE equivalents*.* A comparison with MoCA and MMSE showed that, although both tests did significantly predict group status (MoCA β = 0.29, OR = 1.34, *P* ≤0.001, MMSE β = 0.30, OR = 1.36, *P* ≤0.001), the BMET provided better discrimination (MoCA AUC = 0.77, 95% CI = 0.67–0.87; BMET vs. MoCA χ^2^ = 13.96, *P* ≤0.001; MMSE AUC = 0.70, 95% CI = 0.59–0.81; BMET vs. MMSE χ^2^ = 20.9, *P* ≤0.001; Figure 1). The MoCA and MMSE did not significantly differ between each other in their discriminative ability (χ^2^ = 1.6, *P* = 0.20). Post-hoc analysis comparing the AUCs of the BMET, MoCA, and MMSE when defining cognitive impairment using more stringent criteria (see Methods) resulted in similar results (BMET AUC = 0.95, 95% CI = 0.91–0.99, MoCA AUC = 0.89, 95% CI = 0.83–0.94, MMSE AUC = 0.75, 95% CI = 0.67–0.86).

## Discussion

Building on our previous study demonstrating the effectiveness of the BMET in differentiating patients with SVD from those with Alzheimer’s disease [10], this multicentre evaluation established that the BMET, when administered by non-psychologists, showed good sensitivity and specificity in the detection of CI in patients with SVD. It showed significantly better performance in detecting VCI than either of the two measures commonly used in current clinical practice, the MMSE and the MOCA. The BMET was reliably administered by non-psychologists in a mean time of 13 minutes in the SVD group, making it appropriate for use as a brief screening measure. We collected data allowing construction of population norms which will be useful for implementation of the BMET. Using these, we established an optimal clinical cut-off of 13, at which the BMET showed a high sensitivity (93%) and specificity (76%) for detecting VCI.

This paper demonstrates the sensitivity of the BMET as a tool to screen for VCI in people with SVD, and provides age-normed scaled scores based on a large sample of healthy older adults selected from across the UK. It should be noted that while we compared the BMET to the MoCA and MMSE, there are other tests already available that may have a greater focus of executive functioning. For instance, the CAMCOG [18] includes more measures of executive functioning, but may be too long and cover too many domains of functioning to be a useful screening test in SVD. The INCEO Frontal Screening [19], which although perhaps more useful, does not include the tests which have been shown to me most sensitive to SVD such as the trail-making task or digit-symbol coding [8]. Nevertheless, it would be useful to demonstrate empirically the relative strengths and weaknesses of each of these measures versus the BMET.

The better performance of the BMET compared with the MMSE and MoCA is due to its specific development for this purpose, in contrast with the existing tests which have been developed to detect cortical dementias such as Alzheimer’s disease. In contrast, the BMET focuses on cognitive difficulties that are more prominent in SVD and sub-cortical VCI, namely executive functioning and processing speed [20,21]. While the need for such a measure has been recognised for some time [9], most previously suggested protocols, while sensitive, take too long to administer [8,9] to be useful as a brief assessment in the acute or out-patient clinic settings. Furthermore, by also including tests of orientation and memory, the BMET has the scope to differentiate between SVD with CI and early Alzheimer’s disease, as was shown in our preliminary study [10]. In addition to the BMET’s clinical utility, it also shows promise for use in research studies in which brief measures attuned to the cognitive profile of SVD are needed. Given that we have shown that the BMET has good test-retest reliability, applications could include the use of the BMET in large epidemiological studies or clinical trials which require a large number of patients to have cognitive testing. In the latter, parallel versions are likely to be necessary to reduce learning effects. The importance of using cognitive tests sensitive to the deficit seen in patients with SVD to detect treatment effects in clinical trials has been previously documented [22]. Furthermore, it is particularly important for sensitive neuropsychological screening tools to be implemented in trials involving first-ever lacunar stroke patients who may have subtle cognitive difficulties which may predict latter VCI [5].

Despite evidence that the BMET is a strong predictor of VCI in SVD, there are a number of study limitations that should be considered. Firstly, in order to validate the use of the BMET in a wide range of SVD patients, we included the full range of patients with lacunar stroke from those with isolated lacunar infarcts to multiple lacunar infarcts and from no to severe leukoaraiosis. SVD is a heterogeneous disease and future research should show that the results presented here are generalizable across all its clinical presentations. It has also been suggested that CI is more common in those who have multiple lacunar infarcts [23].

Furthermore, while the BMET demonstrated good internal consistency for the total score and the executive functioning/processing speed subscale, this was substantially lower for the orientation/memory subscale in the SVD group. We suggest that the reason for this is the relative sparing of orientation ability compared to executive functioning/processing speed in those with SVD. Given that this subscale is primarily intended to aid in the differentiation between SVD and Alzheimer’s disease [10], future research is required to investigate the psychometric properties of this subscale in that patient group.

Despite having a relatively large sample size of healthy controls, dividing our sample into age-ranges for the development of scaled scores significantly reduced our individual cell size in the older age ranges (e.g., 85–90), possibly making the scaled scores less reliable. More research focusing on recruiting a larger and more representative sample of those aged >80 years of age will be required. We plan to continue to build our normative sample for the BMET allowing us to refine the scales and conduct additional analyses, including the stratification groups by not only age but also education and gender.

This study would have benefited from a more comprehensive evaluation of neuropsychological performance as an external validator of the presence of cognitive impairment. One future direction would be to assess how accurately the cognitive profile identified by the BMET maps onto a full neuropsychological assessment both acutely and in longitudinal studies to see whether the BMET can be used to predict the later development of VCI. It is important that sensitive neuropsychological screening tools are implemented in trials involving first-ever lacunar stroke patients who may have subtle cognitive difficulties to allow the determination of whether such deficits predict the development of vascular dementia [5].

## Conclusions

In conclusion, we have demonstrated the utility of the BMET as a tool to screen for VCI in patients with SVD, a disease group who make up the majority of cases of CI associated with cerebrovascular disease and who are poorly served by current screening tests. We found that the measure has good sensitivity and specificity and, in this patient group, outperformed two commonly used brief cognitive screening tools. Furthermore, the BMET’s short administration time and good test-retest reliability indicates that it could be effective across a number of clinical and research settings.

## Additional files

Additional file 1:
**Brief memory and executive test subtest descriptions.**
Additional file 2:
**Age-normed cut-off scores for Brief Memory and Executive Test (BMET) subscales and recommended cut-off scores for BMET total score versus the Montreal Cognitive Assessment and Mini Mental State Examination.**

## Abbreviations

AUC: Area under the curve
BMET: Brief Memory and Executive Test
CI: Cognitive impairment
MMSE: Mini Mental State Examination
MoCA: Montreal Cognitive Assessment
NART: National Adult Reading Test
ROC: Receiver Operating Characteristic
SD: Standard deviation
SVD: Small vessel disease
VCI: Vascular cognitive impairment
