# Supplementary material for: The Brief Memory and Executive Test (BMET) for detecting vascular cognitive impairment in small vessel disease: a validation study
Source: BMC Med. 2015 Mar 11;13:51. doi: 10.1186/s12916-015-0290-y (PMC4372040; doi:10.1186/s12916-015-0290-y)
Supplement: Additional file 1: — Brief memory and executive test subtest descriptions. [file 12916_2015_290_MOESM1_ESM.docx]

**Additional File 1**

**BMET Subtest descriptions**

***i. Orientation***

Ten questions concerning time and place orientation and autobiographical facts, such as name and date of birth (max 10 points).

***ii. Letter-number matching***

This test of processing speed requires the participant to use a key to write appropriate numbers into a serious of empty boxes arranged in a grid. The numbers are chosen by seeing a letter above each empty box and finding the corresponding letter within a key at the top of the page which has the letter underneath a designated matching number. There are five letter-number pairs. The grid contains 40 letters entered in a pseudo random order. The score is the total correct numbers entered within 45 seconds (max 40 points).

***iii. Five item repetition***

This task involves working memory and verbal learning procedure. Five words are read aloud by the assessor and the participant repeats them back immediately (in any order). This is repeated twice with a different pseudo random order of presentation. The score is the total correct over the 3 trials (max 15 points).

***iv. Sequencing (Motor, letter, letter-number)***

The task has three levels of difficulty, reflecting increasing executive function load. All three are presented on an A4 page of paper, are preceded by short practice items and involve pencil drawing. M*otor Sequencing* requires tracing over a simple line which connects up a sequence of boxes. *Letter Sequencing* requires joining up pseudo-randomly placed diamonds which contain letters of the alphabet, in alphabetical order, whilst ignoring empty distracter diamonds. *Letter-number Sequencing* requires switching between numbers and letters, for example, ‘1-A-2-B…’. If errors are made, participants are redirected to the previous point and asked to continue from there. Motor and letter sequencing task are each allotted maximum times of 180 seconds, with 300 seconds letter-number sequencing. The score for each test is the time taken to complete each trial.

***v. Five item recall (adjusted for intrusions)***

This is an episodic memory task. Participants are asked to recall the five words from the five-item repetition task. The score is calculated as the total correct minus the total intrusions (max 5 points).

***vi. Five item recognition (adjusted for intrusions)***

This is a cued episodic memory task. The five words from the five-item repetition task are displayed on a page mixed with 20 distracter words. The total score is the total correct minus the false positives (max 5 points).
