# Supplementary material for: The Brief Memory and Executive Test (BMET) for detecting vascular cognitive impairment in small vessel disease: a validation study
Source: BMC Med. 2015 Mar 11;13:51. doi: 10.1186/s12916-015-0290-y (PMC4372040; doi:10.1186/s12916-015-0290-y)
Supplement: Additional file 2: — Age-normed cut-off scores for Brief Memory and Executive Test (BMET) subscales and recommended cut-off scores for BMET total score versus the Montreal Cognitive Assessment and Mini Mental State Examination. [file 12916_2015_290_MOESM2_ESM.docx]

**Additional File 2**. Age-normed cut-off scores for B-MET subscales and recommended cut-off scores for B-MET total score versus the MoCA and MMSE

T1) Number in each age-group within the normative sample

| Group | n |
| --- | --- |
| 40 – 49 | 72 |
| 50 -59 | 71 |
| 60 – 69 | 49 |
| 70 – 74 | 30 |
| 75 – 79 | 37 |
| 80 – 84 | 36 |
| 85- 90 | 8 |

T2) Orientation

| Group | 2 | 1 | 0 |
| --- | --- | --- | --- |
| 40 – 49 | 10 | 9 | ≤ 8 |
| 50 -59 | 10 | 9 | ≤ 8 |
| 60 – 69 | 10 | 9 | ≤ 8 |
| 70 – 74 | 10 | 9 | ≤ 8 |
| 75 – 79 | 10 | 9 | ≤ 8 |
| 80 – 84 | 10 | 9 | ≤ 8 |
| 85- 90 | 10 | 9 | ≤ 8 |

T3) Five Item Repetition

| Group | 2 | 1 | 0 |
| --- | --- | --- | --- |
| 40 – 49 | 15 | 14 | < 14 |
| 50 -59 | ≥ 14 | 13 | < 13 |
| 60 – 69 | ≥ 14 | 13 | < 13 |
| 70 – 74 | ≥ 14 | 13 | < 13 |
| 75 – 79 | ≥ 13 | 11 - 12 | < 11 |
| 80 – 84 | ≥ 13 | 11 - 12 | < 11 |
| 85- 90 | ≥ 11 | 8 - 10 | < 10 |

T4) Letter-Number Matching

| Group | 2 | 1 | 0 |
| --- | --- | --- | --- |
| 40 – 49 | ≥ 28 | 22 -27 | < 22 |
| 50 -59 | ≥ 21 | 14 - 20 | < 14 |
| 60 – 69 | ≥ 18 | 10 – 17 | < 10 |
| 70 – 74 | ≥ 17 | 10 – 16 | < 10 |
| 75 – 79 | ≥ 15 | 8 - 14 | < 8 |
| 80 – 84 | ≥ 15 | 8 - 14 | < 8 |
| 85- 90 | ≥ 15 | 8 - 14 | < 8 |

T5) Motor Sequencing

| Group | 2 | 1 | 0 |
| --- | --- | --- | --- |
| 40 – 49 | ≤ 17 | 18 – 24 | > 24 |
| 50 -59 | ≤ 27 | 28 – 40 | > 40 |
| 60 – 69 | ≤ 30 | 31 – 45 | > 45 |
| 70 – 74 | ≤ 40 | 41 – 62 | > 62 |
| 75 – 79 | ≤ 40 | 41 – 62 | > 62 |
| 80 – 84 | ≤ 50 | 51 – 78 | > 79 |
| 85- 90 | ≤ 50 | 51 – 78 | > 79 |

T6) Letter Sequencing

| Group | 2 | 1 | 0 |
| --- | --- | --- | --- |
| 40 – 49 | ≤ 47 | 48 – 64 | > 64 |
| 50 -59 | ≤ 63 | 64 – 79 | > 80 |
| 60 – 69 | ≤ 63 | 64 – 105 | > 105 |
| 70 – 74 | ≤ 65 | 64 – 105 | > 105 |
| 75 – 79 | ≤ 83 | 84 – 113 | > 113 |
| 80 – 84 | ≤ 89 | 90 – 121 | > 121 |
| 85- 90 | ≤ 97 | 98 – 126 | > 126 |

T7) Number Letter Sequencing

| Group | 2 | 1 | 0 |
| --- | --- | --- | --- |
| 40 – 49 | ≤ 53 | 54 – 72 | > 72 |
| 50 -59 | ≤ 92 | 93 – 134 | > 134 |
| 60 – 69 | ≤ 104 | 105 – 149 | > 149 |
| 70 – 74 | ≤ 134 | 135 – 197 | > 197 |
| 75 – 79 | ≤ 140 | 141 – 204 | > 204 |
| 80 – 84 | ≤ 140 | 141 – 204 | > 204 |
| 85- 90 | ≤ 140 | 141 – 204 | > 204 |

T8) Five Item Memory (Delayed Recall)

| Group | 2 | 1 | 0 |
| --- | --- | --- | --- |
| 40 – 49 | ≥ 3 | 2 | < 1 |
| 50 -59 | ≥ 3 | 2 | < 1 |
| 60 – 69 | ≥ 2 | 1 | 0 |
| 70 – 74 | ≥ 2 | 1 | 0 |
| 75 – 79 | ≥ 2 | 1 | 0 |
| 80 – 84 | ≥ 1 | - | 0 |
| 85- 90 | ≥ 1 | - | 0 |

T9) Five Item Memory (Delayed Recognition)

| Group | 2 | 1 | 0 |
| --- | --- | --- | --- |
| 40 – 49 | ≥ 4 | 3 | < 3 |
| 50 -59 | ≥ 3 | 2 | < 2 |
| 60 – 69 | ≥ 3 | 2 | < 2 |
| 70 – 74 | ≥ 3 | 2 | < 2 |
| 75 – 79 | ≥ 3 | 2 | < 2 |
| 80 – 84 | ≥ 3 | 2 | < 2 |
| 85- 90 | ≥ 2 | 1 | 0 |

T10) Sensitivity and Specificity of B-MET cut-off scores versus the MMSE and MoCA

| B-MET | | | MMSE | | | MoCA | | |
| --- | --- | --- | --- | --- | --- | --- | --- | --- |
| Cut off | Sensitivity | Specificity | Cut off | Sensitivity | Specificity | Cut off | Sensitivity | Specificity |
| 10 | 77 % | 95 % | 24 | 26 % | 93 % | 24 | 77 % | 66 % |
| 11 | 85 % | 88 % | 25 | 33 % | 90 % | 25 | 77 % | 50 % |
| 12 | 88 % | 84 % | 26 | 41 % | 83 % | 26 | 92 % | 36 % |
| 13 | 92 % | 76 % | 27 | 56 % | 72 % | 27 | 96 % | 25 % |
| 14 | 96 % | 60 % | 28 | 70 % | 58 % | 28 | 100% | 14 % |
| 15 | 100% | 42 % | 29 | 88 % | 35 % | 29 | 100% | 4 % |
